# The Impact of Air Pollution on Intestinal Microbiome of Asthmatic Children: A Panel Study

**DOI:** 10.1155/2020/5753427

**Published:** 2020-11-05

**Authors:** Ping Zheng, Bei Zhang, Kexing Zhang, Xifang Lv, Qiang Wang, Xuetao Bai

**Affiliations:** ^1^National Institute of Environmental Health, Chinese Center for Disease Control and Prevention, Beijing 100050, China; ^2^Xinwu District Center for Disease Control and Prevention, Wuxi 214000, China

## Abstract

Air pollution could impact on the alteration of intestinal microbiome. Maturation of intestinal microbiome in early life played an important role in the development of allergic diseases, including asthma. Recent studies presented an increase in the evidence of association between the shift of gut microbiota and asthma. This article is aimed at exploring whether the alteration in the intestinal microbiome triggered by a short wave of air pollution could influence the colonization of bacteria that have been related to the immunological mechanisms of the asthma attack. The impact of air pollution on intestinal microbiome was assessed by longitudinal comparison. Fecal samples were collected twice for twenty-one children in clean and smog days, respectively, including eleven asthmatic children and ten healthy children. Intestinal bacteria were discriminated by using the method of 16S rRNA gene sequence. The results showed that the composition of intestinal microbiome changed between clean and smog days among all children (PERMANOVA, *P* = 0.03). During smog days, *Bifidobacteriaceae*, *Erysipelotrichaceae*, and *Clostridium sensu stricto 1* decreased, and *Streptococcaceae*, *Porphyromonadaceae*, *Rikenellaceae*, *Bacteroidales S24-7 group*, and *Bacteroides* increased in asthmatic children (Wilcoxon test, *P* < 0.05), while *Fusicatenibacter* decreased and *Rikenellaceae* and *Terrisporobacter* increased in healthy children (Wilcoxon test, *P* < 0.05). After controlling for food consumption, the relative abundance of some bacteria belonging to *Firmicutes* negatively associated with concentration of PM_2.5_, PM_10_, NO_2_, and SO_2_ (multiple linear regression, *P* < 0.05). This study demonstrated that short wave of air pollution had an impact on the intestinal microbiome of asthmatic children. Intestinal bacteria, which have been related to immunological mechanisms of asthma attack, were also found to be associated with air pollution. This finding suggested that a short wave of air pollution may trigger asthma by impacting on intestinal bacteria.

## 1. Introduction

Asthma is a heterogeneous disease. Air pollution was found as one of the important triggers or potential risk factors of asthma [1–5]. Existing research presented that air pollution might contribute to the development and exacerbation of asthma through four main mechanisms [6], including oxidative stress and damage [7–10], airway remodeling [11, 12], inflammatory pathways and immunological responses [13–16], and enhancement of respiratory sensitization to aeroallergens [17–19], while the existing studies have not been consistent on every mechanism. The mechanisms of air pollutants triggering asthma were not completely clear yet.

The alteration of intestinal microbiome was reported to be associated with the rising of allergic diseases, including asthma. The “hygiene hypothesis” firstly suggested a link between microbes and allergy [20]. Then, many studies showed that maturation of intestinal microbiome in early life played an important role in the development of asthma [21–24]. As the development of the next-generation sequence, the composition of intestinal microbiome was found significantly different in asthmatics [25–27]. The mechanisms of intestinal microbiome impact on asthma are involved with the activities of regulatory T cells (Tregs). Tregs have a central role in the suppression of inflammatory and allergic responses, which can be induced by symbiotic microbes [28–30]. Intestinal microbiome may modulate Tregs in immune function by producing local and systemic mediators, such as lipopolysaccharides, peptidoglycans, and short-chain fatty acids, and impact on asthma development by the gut-lung axis [31–36]. Furthermore, air pollutants including particulate matter and nitrogen dioxide were shown to alter the composition of intestinal microbiome [37–39].

Based on the above findings, we could get a clue about air pollution, intestinal microbiome, immunological responses, and asthma attack. Thus, we assumed that air pollution might trigger the allergy mechanism of asthmatic children through impacting on the intestinal microbiome. If it does so, the composition of intestinal microbiome in asthmatic children should change before and after air pollution days. This article is aimed at studying the alteration of intestinal microbiome in asthmatic children in smog days, to further explore whether dysbiosis of the intestinal microbiome induced by smog was associated with the immunological mechanisms of asthma attack.

## 2. Materials and Methods

### 2.1. Study Population

This study was part of a panel study funded by the National Natural Science Foundation of China. The panel study is aimed at studying the epigenetic modification mechanisms of black carbon on nonatopic asthma. In the investigation process, we found that more allergic children with asthma came to the hospital in smog days. Based on the connection of asthma, intestinal microbiome, and air pollution, we conducted this exploratory study. Participants were recruited from all 176 children enrolled in the panel study. All participants were aged 5-12 years old. They all lived in Beijing. Children met the criteria of sample collection, and those who agreed to join in this study would offer fecal samples. We calculated the sample size according to the method for paired data. Sample size was required to find significant difference in alpha diversity. We took 0.8 for statistical power, 0.05 for significance level, and 0.7 for effect sizes. The sample size should be no less than 18 participants. This study was approved by the ethical review committee of the Institute for Environmental Health and Related Product Safety, Chinese Center for Disease Control and Prevention (201501). All participants and their legal guardians signed the written informed consent.

### 2.2. Health Data Collection

Status on asthma and wheezing for all participants were collected by using a questionnaire based on the ISAAC (International Study of Asthma and Allergies in Childhood) questionnaires. Then, asthmatic children were further diagnosed according to the Global Initiative for Asthma (http://ginasthma.org) by physicians from the Children Hospital of Capital Institute of Pediatrics in Beijing. Briefly, asthma was confirmed as repeated attacks of respiratory symptom (wheezing, shortness of breath, chest tightness, and cough), together with variable expiratory airflow limitation. The variable expiratory airflow limitation was confirmed by the reduction of lung function. Lung function of asthmatic children was measured by physicians in this study. In addition, the frequency of food consumption in the sampling interval was investigated by a questionnaire based on NHANES (National Health and Nutrition Examination Survey) food questionnaire. Food consumption mainly included vegetables, mushrooms, fruit, and eggs. Carbohydrate intake was defaulted to no difference, as all children share similar carbohydrate consumption in their diet based on the local food traditions.

### 2.3. Definition of Clean and Smog Days

Fecal samples were collected twice for each participant before and after smog days in 2017. The air quality for sampling days should meet the following criteria. The first sampling was performed in clean days; the mean AQI (Air Quality Index) of the five days before sampling day should be all less than 100. The second sampling was performed in smog days; the mean AQI of the five days before sampling should be all more than 100. A limit of 100 for AQI was taken according to the Technical Regulation on Ambient Air Quality Index (on trial), Ministry of Environmental Protection, China. AQI > 100 was defined as mild air pollution. In such weather, the symptoms of the susceptible population will be aggravated. The environmental data of air quality were from the China National Environmental Monitoring Centre (http://www.cnemc.cn). Exposure data for each child was achieved from the monitoring station nearest to his or her house; the average distance was 4.5 ± 2.0 kilometers.

### 2.4. Fecal Sample Collection

Fecal sample collection was required for all participants. All participants were asked to meet the following criteria: no antibiotic use history and vaccination history in the past one month, no injury, and no diarrhea in the past two weeks. In addition, girls were not in the period of the menstrual cycle when offering feces. Fecal samples were collected with sterile plastic tubes. Before detecting, the samples were stored at a temperature of -80°C.

### 2.5. DNA Extracting and Sequencing

Assessment of the intestinal microbiome in fecal samples was carried out at Novogene Bioinformatics Technology Co. Total genome DNA was extracted from 0.5 g feces samples using a modified CTAB (Cetyltrimethylammonium Bromide) method [40, 41]. The V4 region of 16S rRNA genes was amplified by using the specific primers of 515F (GTGCCAGCMGCCGCGGTAA) and 806R (GGACTACHVGGGTWTCTAAT) with the barcodes. PCR reactions were performed using Phusion® High-Fidelity PCR Master Mix (New England Biolabs) according to the manufacturer's guidelines. Sequencing libraries for the PCR products were generated using TruSeq® DNA PCR-Free Sample Preparation Kit following the manufacturer's recommendations. Then, library quality was assessed on the Qubit 2.0 Fluorometer (Thermo Scientific) and Agilent Bioanalyzer 2100 system. Paired-end sequence was performed on the Illumina HiSeq 2500 (Illumina Inc., CA, USA).

### 2.6. Sequencing Data Analysis

The original data was assigned to each sample according to the barcode. The barcodes and primers were removed from the original data by Cutadapter. Then, paired-end reads from the original DNA fragments were merged using FLASH [42]. Quality filtering on the raw tags was performed by QIIME (V1.9.1) according to the quality-controlled process [43]. The filtered tags were compared with Gold database using the UCHIME algorithm [44] to detect chimera sequences, and then chimera sequences were removed [45], and the effective tags were finally obtained. The sequences analyses were clustered by the Uparse software (V7.0.1001) [46]. Sequences with ≥97% similarity were assigned to the same OTUs (operational taxonomic units). Representative sequence for each OTU was screened for further annotation. Taxa annotation was performed by using the method of Mothur according to SSU rRNA database of SILVA with a threshold of 0.8 to 1 [47]. Normalization of the OTU abundance was achieved using a standard number corresponding to the sample with the least sequence number.

### 2.7. Verification Test

To further demonstrate the correlation between the dysbiosis of intestinal microbiome triggered by air pollution and asthma attack, we performed a verification test on asthmatic children. Fractional exhaled nitric oxide (FeNO) was measured by physicians for evaluation of respiratory tract inflammation of asthmatic children, which was correlated with the allergic status of asthma attack. Then, the correlations between FeNO and intestinal bacteria associated with air pollution were analyzed to verify the function of the intestinal bacteria on asthma attack.

### 2.8. Statistical Analysis

Statistical analysis for sequence data was performed based on the normalized data. Chao1 and Shannon parameters were calculated by the QIIME software to estimate alpha diversity. Both weighted and unweighted [48, 49] unique fraction metric (UniFrac) distances were calculated by QIIME to estimate beta diversity. Analyses for significance of bacteria abundance between asthmatic and healthy children were tested using Wilcoxon test by R software (V 3.4.3). Adonis function in the vegan package of R software was used to conduct permutational multivariate analysis of variance (PERMANOVA) for intestinal microbiome composition. Bacteria with statistical and biological significance between clean and smog days were evaluated by the Linear Discriminant Analysis (LDA) of effect size (LEfSe) [50]. The associations between intestinal bacteria, air pollutants, and FeNO were analyzed using multiple linear regression [51]. A two-tailed *P* value of <0.05 was considered significant.

## 3. Results

### 3.1. Characteristics of Study Population

In total, 21 participants met the criteria of sample collection and offered samples in the two sampling time, including 11 asthmatic children (6 boys and 5 girls) and 10 healthy children (7 boys and 3 girls). The average age of the participants was 7.8 ± 1.5 years old. The average value of BMI (Body Mass Index) was 17.1 ± 2.1 kg/m^2^. All participants have mild asthma. Their forced expiratory volume in 1 second (FEV1) was higher than 70%. During sample collection time, they were at stable period of asthma, and they did not orally take any drugs. A total of 36 asthmatic children (21 boys and 15 girls) were enrolled in the verification test. Their average age was 8.0 ± 2.1 years old. The average value of BMI was 17.1 ± 3.1 kg/m^2^.  Table 1 lists the descriptive statistics of participants.

### 3.2. Environmental Data

The 5-day average AQI in clean days was 73. The 5-day average AQI during smog days was 120. Concentrations of all pollutants were higher in smog days. The dominant pollutant in smog days was PM_2.5_. All PM_2.5_ during smog days exceeded the WHO guideline of 24 h mean limits (25 *μ*g/m^3^).  Table 2 shows the concentrations of ambient air pollutants in clean days and smog days.

### 3.3. Sequence Data of Intestinal Microbiome

Sequence data of 42 samples from 21 children were analyzed to describe the changes of microbial community between clean and smog days. In the 42 samples, a mean of 71358 tags, ranging from 59193 to 99554, was generated from the V4 hypervariable region of 16S rRNA gene. A mean of 506 ± 195 OTUs was identified. The average value of OTUs was 480 ± 101 (asthmatic children: 537 ± 93; healthy children: 416 ± 66) in clean days and 533 ± 261 (asthmatic children: 511 ± 75; healthy children: 582 ± 380) in smog days.

### 3.4. Comparison of Bacteria Diversity

**Figure 1:**
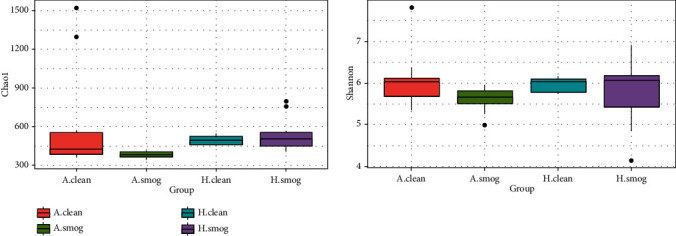
Chao1 and Shannon parameters between clean and smog days by group. A.clean: asthmatic children during clean days; A.smog: asthmatic children during smog days; H.clean: healthy children during clean days; H.smog: healthy children during smog days.

**Figure 2:**
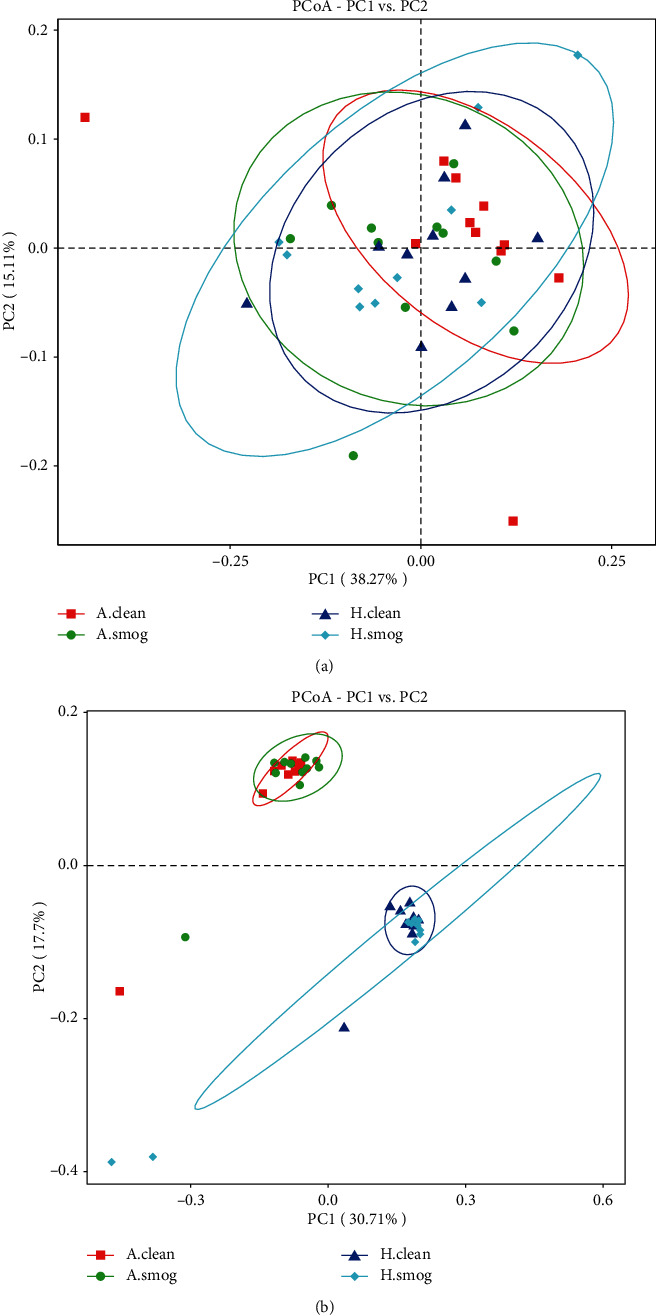
PCoA plot with (a) weighted and (b) unweighted UniFrac distance. It covered all samples. A.clean: asthmatic children during clean days; A.smog: asthmatic children during smog days; H.clean: healthy children during clean days; H.smog: healthy children during smog days.

As a panel study, we assessed the impact of air pollution on intestinal microbiome among all children firstly. Chao1 and Shannon parameters as measures of alpha diversity were not significantly different between clean and smog days (Wilcoxon test, *P* > 0.05).  Figure 1 shows the Chao1 and Shannon parameters of the intestinal microbiome among asthmatic children and healthy children in clean and smog days. PCoA analysis as a measure of beta diversity was conducted based on weighted and unweighted UniFrac distance.  Figure 2 shows the difference of microbial composition between asthmatic and healthy children in clean and smog days. Some discriminations were shown in the plot. Among all children, PERMANOVA based on unweighted UniFrac distance confirmed the changes of microbial composition between clean and smog days (weighted: *R*^2^ = 0.02, *P* = 0.61; unweighted: *R*^2^ = 0.07, *P* = 0.03). The results showed that the composition of intestinal microbiome was altered between clean and smog days.

### 3.5. Comparison of Bacteria Relative Abundance

**Figure 3:**
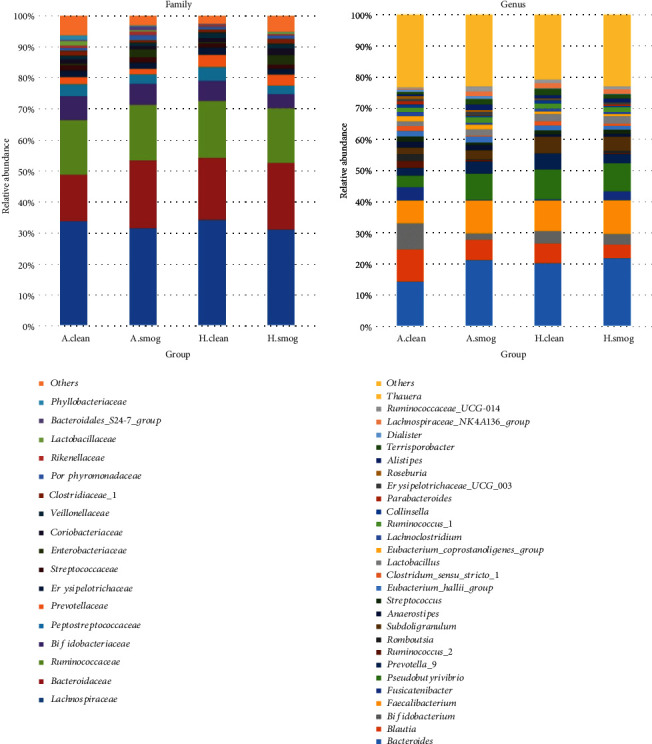
Relative abundance difference of families and genera between clean and smog days. The taxa were shown in different colors; the height of bars represents relative abundance. A.clean: asthmatic children during clean days; A.smog: asthmatic children during smog days; H.clean: healthy children during clean days; H.smog: healthy children during smog days.

We analyzed the changes of bacterial relative abundance between clean and smog days in asthmatic and healthy children, respectively. We analyzed the bacterial relative abundance at the level of phylum, family, and genus. At the phylum level, *Firmicutes*, *Bacteroidetes*, *Actinobacteria*, and *Proteobacteria* were the main taxa, which accounted for more than 99.0% in each group. However, they did not show significant difference between clean and smog days (Wilcoxon test, *P* > 0.05). At the family and genus levels, we analyzed taxa with relative abundance over 1% at least in one group. Seventeen taxa of family and twenty-eight taxa of genus were identified.  Figure 3 shows the relative abundance of them.

Table 3 presents bacteria with statistical difference between clean and smog days at the family and genus levels. Six families showed significant changes in asthmatic children (Wilcoxon test, *P* < 0.05); half of them belong to the *Bacteroidetes* phylum. Only one family (*Rikenellaceae*) showed significant changes in healthy children (Wilcoxon test, *P* < 0.05). At the genus level, *Clostridium sensu stricto 1* and *Bacteroides* significantly changed in asthmatic children between clean and smog days. *Fusicatenibacter* and *Terrisporobacter* significantly changed in healthy children between clean and smog days.

### 3.6. Result of LEfSe

**Figure 4:**
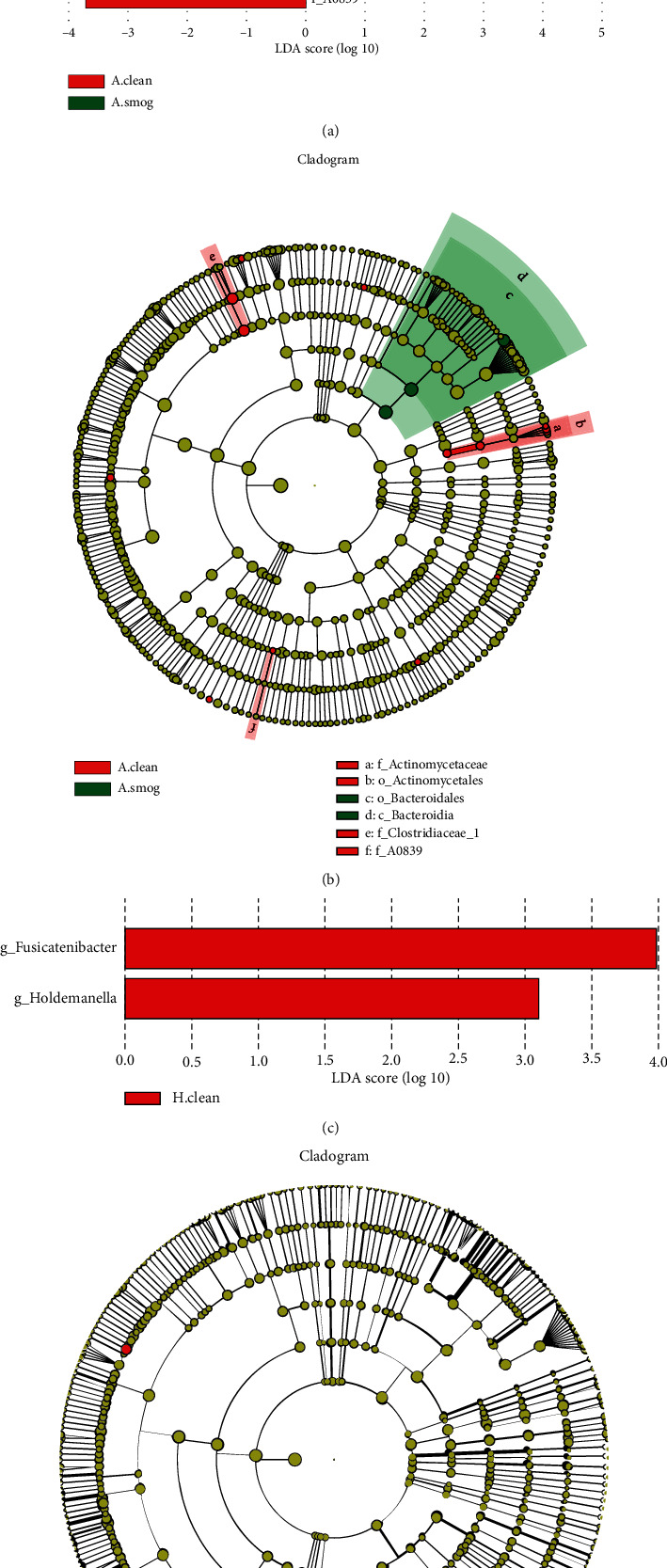
Bacteria with statistical significance between clean and smog days analyzed by LEfSe. (a) LDA score for asthmatic children, (b) cladogram for asthmatic children, (c) LDA score for healthy children, and (d) cladogram for healthy children. The graph showed the LDA scores obtained from linear regression analysis of the significant microorganism groups. It distinguished the microbial communities of each group with LDA > 3 and the *P* value < 0.05. In the cladogram, the circle which radiated from the inside to the outside represented the classification level from the phylum to species. Each dot represented a taxon. Red dots corresponded to taxa with significant difference in clean days. Green dots corresponded to taxa with significant difference in smog days. A.clean: asthmatic children during clean days; A.smog: asthmatic children during smog days; H.clean: healthy children during clean days; H.smog: healthy children during smog days.

For asthmatic children, LEfSe detected that class of *Bacteroidia*, its order *Bacteroidales*, and its species *Bacteroides uniformis* were dominant in smog days; bacteria from *Clostridiales* order (*Clostridiaceae 1*, *Clostridium sensu stricto 1*, and *Peptoclostridium*) and *Actinomycetales* order (*Actinomycetaceae*, *Actinomyces odontolyticus*, *Actinomyces naeslundii*, and *Actinomyces* sp. *oral clone GU009*) were dominant in clean days (Figures 4(a) and 4(b)). The finding reflected that these bacteria from *Clostridiales* order and *Actinomycetales* order decreased in smog days.

For healthy children, *Fusicatenibacter* and *Holdemanella* were richer in clean days (Figure 4(c)). No bacteria with statistical and biological significance were found in smog days.

Other taxa with differences detected by LEfSe, including *Gemmatimonas aurantiaca*, *Lewinella*, *Propionivibrio*, *Streptococcus anginosus*, and *A0839*, all with a low relative abundance (<0.01%), may have little impact on host health.

### 3.7. Association between Intestinal Microbiome and Air Pollutants

We analyzed the association between air pollutants and taxa with significant changes based on the environmental data of smog days by using the method of multiple linear regression analysis. Dietary intakes were controlled because there was no other influence factor that changed during the interval of the two samplings, except for dietary intakes.  Table 4 shows the regression results of the association between ambient air pollutants and intestinal microbiome. After controlling for dietary intake, eight taxa showed associations with air pollutants. *Bifidobacteriaceae* and *Enterobacteriaceae* were positively associated with air pollutants; the others were all negatively associated with air pollutants. Among the eight taxa, four taxa were associated with PM_10_, five taxa were associated with PM_2.5_, six taxa were associated with NO_2_, seven taxa were associated with SO_2_, and no taxon was associated with O_3_.

### 3.8. Verification Test

We assessed the associations between FeNO concentration of asthmatic children and the intestinal bacteria, which were associated with air pollution, by multiple linear regression analysis. Age, sex, and BMI were controlled. We found that the relative abundance of *Enterobacteriaceae* significantly associated with FeNO concentration of asthmatic children (Table 5).

## 4. Discussion

In this article, the result of beta diversity analysis for intestinal bacteria showed that the composition of intestinal microbiome significantly changed in correlation with air pollution exposure (unweighted UniFrac distance: *R*^2^ = 0.07, *P* = 0.03), which was in agreement with previous studies of the relation between air pollution and gut microbiome [37–39]. The unweighted UniFrac distance suggested a difference in qualitative measures of intestinal bacteria between clean and smog days [49]. Human studies have shown that mucociliary transport of inhaled particulate matter (PM) is quickly cleared from the lungs and into the intestine. Furthermore, pollutant PM contaminates both human's food and water supply in significant amounts. It has been estimated that 10^12^~10^14^ particles are ingested per day by an individual [38, 52–54]. As such, air pollution might carry out an important impact on intestinal microbiome.

Then, we assessed the changes of bacterial relative abundance stratified by asthmatic and healthy children. The results showed that 11 taxa at the family and genus levels were found changing in relative abundance between clean and smog days. Five of them belonged to *Firmicutes* and five of them belonged to *Bacteroidetes*. *Firmicutes* and *Bacteroidetes* should play a major role in asthma attack in smog days.

We found that some bacteria with significant changes in this study may be correlated with immunological mechanisms of asthma attack. First, the class of *Clostridia*, as a member of *Firmicutes*, was an important taxon affected by smog. *Clostridia* could produce propionic acid or butyrate to induce Treg production, which has a central role in the suppression of allergic responses correlated with the development of asthma [29, 30]. *Clostridium leptum* belonging to *Clostridia* was reported decreased in asthmatics [55]. In our study, the family of *Clostridiaceae 1* and genus of *Clostridium sensu stricto 1* significantly decreased among asthmatic children in smog days. They are members of *Clostridia*. Their decrease may be related to asthma attack in air pollution. Another important taxon we found was *Bacteroidetes*. More bacteria belonging to the *Bacteroidetes* phylum significantly increased in asthmatic children during smog days. LEfSe detected that the class of *Bacteroidia*, its order *Bacteroidales*, and its species *Bacteroides uniformis* were dominant in smog days in asthmatic children. Kirjavainen et al.'s study showed that serum total IgE concentration correlated directly with *Bacteroides* counts in infants with high sensitivity for allergy [56]. According to the article by Vael et al. [57], *Bacteroides fragilis* colonization at an early age was an indicator of possible asthma later in life. However, *Bacteroides fragilis* monocolonization was reported to correct CD4^+^ T cell deficiency in spleens of germ-free mice [58]. Therefore, the result suggested that *Bacteroidetes* may play a role in the asthma attack in air-polluted days, but how they work needs further study.

We also found that relative abundance of some intestinal bacteria was associated with air pollutants. Though some correlations were not very strong, we thought the findings were valuable. Among the taxa correlated with air pollutants, all taxa belonging to *Firmicutes* showed negative correlation with air pollutants. According to Fujimura et al.'s study [59], *Firmicutes* was a protected factor for asthma. Bacteria belonging to *Firmicutes* were reported decreasing in asthmatics by some other studies [60, 61]. Our finding suggested that air pollutants may stimulate asthma by weakening the protective effects of *Firmicutes*.

In addition, *Rikenellaceae* was the only taxon showing an increase both in asthmatic and healthy children in smog days. It may be worthy of further studies on the correlation with air pollution.

At last, we carried out a verification test on the relation of gut microbiome with asthmatics' clinical parameter (FeNO). We found that concentration of FeNO varied with the relative abundance of *Enterobacteriaceae*, *Lactobacillus*, and *Clostridium sensu stricto 1*. In particular, FeNO was significantly associated with *Enterobacteriaceae*. Furthermore, *Enterobacteriaceae* is a family from *Proteobacteria*. Previous studies showed that *Proteobacteria* was higher in the airway of asthmatic subjects [61, 62]. According to the theory of gut-lung axis, surviving gut bacteria or fragments of dead bacteria may migrate from the mesenteric lymphatic system to have access to pulmonary circulation [63]. This finding confirmed that the shift of intestinal microbiome triggered by a short wave of air pollution plays an important role in asthma attack.

On the other hand, a limitation of this study was that the sample size was small. Based on the alpha diversity, we could not conclude a significant change influenced by smog. It may be limited by the sample size. However, some other studies also did not find significant changes about alpha diversity in asthmatics [26]. Changes in intestinal microbiome alpha diversity need to be further explored.

## 5. Conclusions

Air pollution had an impact on the intestinal microbiome of asthmatic children. Intestinal microbiome changed both in asthmatic and healthy children after air pollution days. Some bacteria, which have been related to immunological mechanisms of asthma attack, were correlated with air pollution. This finding suggested that a short wave of air pollution may trigger asthma by impacting on intestinal bacteria, which may help to further study on the mechanism of asthma triggered by air pollution.

## Figures and Tables

**Table 1:** Descriptive statistics of participants.

Characteristics	*n*	Min	P25th	P50th	P75th	Max
High-fiber food intake frequency^1^ (times)						
All	21	3	4	5	6	11
Asthmatic children	11	3	4.5	5	6	8
Healthy children	10	3	3.3	4.5	6	11
High-protein food intake frequency^2^ (times)						
All	21	2	4	4	4	4
Asthmatic children	11	3	3.5	4	4	4
Healthy children	10	2	4	4	4	4
Lung function of asthmatic children (%)						
FEV1	11	75.1	81.9	92.4	106.2	115.0
FVC^3^	11	82.4	88.8	96.0	106.6	107.1
PEF^4^	11	57.9	73.5	86.7	103.8	106.0
MMEF75/25^5^	11	43.7	52.8	67.5	82.8	104.6
FeNO of asthmatic children for verification test (ppb)	36	7.0	15.0	20.0	40.3	133.0

^1^High-fiber food included vegetables, mushrooms, and fruit. ^2^High-protein food included eggs and duck eggs. ^3^FVC: forced vital capacity. ^4^PEF: peak expiratory flow. ^5^MMEF: maximal midexpiratory flow. Data in this table were presented in percentiles.

**Table 2:** Concentrations of ambient air pollutants (percentile, *μ*g/m^3^).

Pollutants^1^	Clean days					Smog days				
	Min	25th	50th	75th	Max	Min	25th	50th	75th	Max
PM_2.5_	16.3	26.3	29.0	37.3	84.7	38.3	38.3	60.3	84.7	149.3
PM_10_	17.7	53.7	55.3	76.7	118.0	68.3	68.3	102.0	138.0	162.0
NO_2_	17.7	33.3	43.3	44.7	55.0	25.7	25.7	44.7	48.7	69.7
SO_2_	2.3	4.0	6.7	10.0	10.3	4.0	4.0	8.0	10.0	18.0
O_3_	40	40	48	77	94	30	62	119	151	177

^1^Concentration of PM_2.5_, PM_10_, NO_2_, and SO_2_ was the 3-day moving average concentration; ozone (O_3_) was the maximum daily 8-hour mean concentration.

**Table 3:** Taxa with significant changes at family and genus levels between clean and smog days (Wilcoxon test).

Taxa	Affiliated phylum	Mean relative abundance (%)		*P* value
		Clean days	Smog days	
Asthmatic children				
Family *Bifidobacteriaceae*	*Actinobacteria*	7.86	6.79	0.04
Family *Erysipelotrichaceae*	*Firmicutes*	2.07	1.95	<0.01
Family *Streptococcaceae*	*Firmicutes*	1.82	1.92	0.04
Family *Porphyromonadaceae*	*Bacteroidetes*	1.10	1.63	<0.01
Family *Rikenellaceae*	*Bacteroidetes*	0.69	1.04	<0.01
Family *Bacteroidales S24-7 group*	*Bacteroidetes*	0.31	1.15	0.03
Genus *Clostridium sensu stricto 1*	*Firmicutes*	1.66	0.28	0.01
Genus *Bacteroides*	*Bacteroidetes*	14.13	21.54	0.05
Healthy children				
Family *Rikenellaceae*	*Bacteroidetes*	0.53	0.66	0.04
Genus *Fusicatenibacter*	*Firmicutes*	4.32	0.26	0.03
Genus *Terrisporobacter*	*Firmicutes*	0.71	1.90	0.03

**Table 4:** Regression results of the association between ambient air pollutants and intestinal bacteria.

Bacteria impacted^1^	Pollutants^2^	Adjusted coefficients^3^	*P* value
Phylum *Actinobacteria*			
Family *Bifidobacteriaceae*	PM_10_	1.60 × 10^−3^	0.013
	PM_2.5_	3.90 × 10^−4^	0.003
	NO_2_	1.19 × 10^−3^	0.004
	SO_2_	4.06 × 10^−3^	0.010
Phylum *Proteobacteria*			
Family *Enterobacteriaceae*	PM_10_	1.60 × 10^−3^	0.013
	PM_2.5_	1.02 × 10^−3^	0.022
	NO_2_	3.70 × 10^−3^	0.008
	SO_2_	1.35 × 10^−2^	0.011
Family *Phyllobacteriaceae*	PM_10_	−1.72 × 10^−5^	0.002
	PM_2.5_	−1.13 × 10^−5^	0.002
	NO_2_	−2.57 × 10^−5^	0.037
	SO_2_	−1.47 × 10^−4^	0.001
Phylum *Bacteroidetes*			
Family *Porphyromonadaceae*	SO_2_	−8.33 × 10^−4^	0.014
Phylum *Firmicutes*			
Family *Clostridiaceae 1*	NO_2_	−4.73 × 10^−4^	0.024
Genus *Lactobacillus*	PM_10_	−1.04 × 10^−4^	0.014
	PM_2.5_	−7.78 × 10^−5^	0.005
	NO_2_	−2.01 × 10^−4^	0.035
	SO_2_	−8.48 × 10^−4^	0.015
Genus *Clostridium sensu stricto 1*	PM_2.5_	−6.70 × 10^−5^	0.025
	NO_2_	−2.39 × 10^−4^	0.008
	SO_2_	−7.80 × 10^−4^	0.031
Species *Eubacterium dolichum*	SO_2_	−9.09 × 10^−4^	0.007

^1^Dependent variable; ^2^independent variable; ^3^single pollutant model, adjusted by dietary intake. Only taxa with significant difference (*P* < 0.05) were shown.

**Table 5:** Regression results of the association between FeNO of asthmatic children and intestinal bacteria.

Dependent variable	Independent variable	Mean relative abundance (%)	Adjusted coefficients	*P* value
FeNO (ppb)	*Bifidobacteriaceae*	9.45	-0.47	0.349
	*Enterobacteriaceae*	0.74	9.78	0.002^∗∗^
	*Phyllobacteriaceae*	0.45	0.09	0.962
	*Porphyromonadaceae*	1.33	1.24	0.677
	*Clostridiaceae 1*	1.73	3.77	0.171
	*Lactobacillus*	0.67	-15.66	0.092^∗^
	*Clostridium sensu stricto 1*	1.64	4.99	0.083^∗^
	*Eubacterium dolichum*	0.01	-781.50	0.165

^∗∗^ *P* < 0.05 and ^∗^*P* < 0.1.

## Data Availability

The datasets generated or analyzed during the current study could be available in the National Center for Biotechnology Information (https://www.ncbi.nlm.nih.gov/), with BioProject ID PRJNA532496.
